# Spatial hearing of normally hearing and cochlear implanted children

**DOI:** 10.1016/j.ijporl.2011.01.002

**Published:** 2011-04

**Authors:** John Murphy, A. Quentin Summerfield, Gerard M. O’Donoghue, David R. Moore

**Affiliations:** a ENT Department, Queen's Medical Centre, Nottingham NG7 2UH, UK; b Department of Psychology, University of York, York Y010 5DD, UK; c National Biomedical Research Unit in Hearing, Ropewalk House, 113 The Ropewalk, Nottingham NG1 5DU, UK; d MRC Institute of Hearing Research, University Park, Science Road, Nottingham NG7 2RD, UK

**Keywords:** Unilateral cochlear implants, Bilateral cochlear implants, Lateral release, Sound localization, Head movements

## Abstract

**Objective:**

Spatial hearing uses both monaural and binaural mechanisms that require sensitive hearing for normal function. Deaf children using either bilateral (BCI) or unilateral (UCI) cochlear implants would thus be expected to have poorer spatial hearing than normally hearing (NH) children. However, the relationship between spatial hearing in these various listener groups has not previously been extensively tested under ecologically valid conditions using a homogeneous group of children who are UCI users. We predicted that NH listeners would outperform BCI listeners who would, in turn, outperform UCI listeners.

**Methods:**

We tested two methods of spatial hearing to provide norms for NH and UCI using children and preliminary data for BCI users. NH children (*n* = 40) were age matched (6–15 years) to UCI (*n* = 12) and BCI (*n* = 6) listeners. Testing used a horizontal ring of loudspeakers within a booth in a hospital outpatient clinic. In a ‘lateral release’ task, single nouns were presented frontally, and masking noises were presented frontally, or 90° left or right. In a ‘localization’ task, allowing head movements, nouns were presented from loudspeakers separated by 30°, 60° or 120° about the midline.

**Results:**

Normally hearing children improved with age in speech detection in noise, but not in quiet or in lateral release. Implant users performed more poorly on all tasks. For frontal signals and noise, UCI and BCI listeners did not differ. For lateral noise, BCI listeners performed better on both sides (within ∼2 dB of NH), whereas UCI listeners benefited only when the noise was opposite the unimplanted ear. Both the BCI and, surprisingly, the UCI listeners performed better than chance at all loudspeaker separations on the ecologically valid, localization task. However, the BCI listeners performed about twice as well and, in two cases, approached the performance of NH children.

**Conclusion:**

Children using either UCI or BCI have useful spatial hearing. BCI listeners gain benefits on both sides, and localize better, but not as well as NH listeners.

## 1 Introduction

Spatial hearing facilitates the ability of a listener to perform in complex listening environments. It refers to a listener's ability to receive, process and utilize directionally specific auditory signals from the two ears, working both independently and in concert [1,2]. In typical, complex listening conditions, when each ear is exposed to a different amalgamation of target signal and noise, a binaural listener can favour the ear with the higher signal-to-noise ratio [SNR]. A high SNR at a single ear can be generated by directionally-sensitive amplification by the outer ear resulting, for example, from a signal placed directly on the ‘acoustic axis’ (about 60° from the midline [3]) of that ear, as well as benefiting from the additional information available to the binaural listener. This ‘monaural listening’ is particularly useful to people with a single functional ear or a large binaural imbalance in sensitivity. For longer signals, these listeners can move their head to align their better hearing ear with the sound source.

Binaural hearing uses several additional mechanisms [4] that improve sound localization and enhance signal detection and segregation in general. Auditory signals received by both ears may be summed, leading to better detection and an increase in loudness relative to a single ear. Binaural localization uses physical differences between the level, onset timing and ongoing phase of sounds arriving at each ear to construct, in the brain, a representation of auditory space. These differences occur for sounds anywhere except the midline of a listener's head. When noise is introduced, and the signal and noise are spatially separated or otherwise interaurally incoherent, a combination of these binaural cues results in ‘unmasking’ (‘squelch’ [5]), making the signal more detectable than would be the case for a single ear.

In hearing impaired people, spatial hearing is dependent on the level and laterality of the hearing loss [6] and on intervention with hearing instruments. Mild to severely hearing impaired people typically receive two hearing aids and can thus benefit from binaural enhancement and at least some binaural interaction [7]. For profoundly deafened individuals, a single cochlear implant [8] has generally been used for the restoration of hearing. Although a unilateral CI (UCI) can produce excellent speech recognition [9], spatial hearing is still severely compromised [10]. Recognition of the important contribution that impaired spatial hearing makes to auditory handicap (e.g. [11]) was one of the leading factors supporting the introduction of bilateral CIs (BCIs). BCIs were thought to be especially important in children, where very early UCI has been shown to be of great benefit for speech perception by prelingually deaf users [12].

Two of the largest studies [8,13] reporting spatial hearing in paediatric BCI listeners found speech perception in noise to be better when using two than when using one CI. However, neither study compared the performance of BCI listeners with a UCI group whose habitual listening condition was monaural only. That requirement was recently met by Lovett et al. [14] who found that 30 BCI children performed significantly more accurately than 20 UCI children on sound localization and speech perception in noise.

For sound localization, Litovsky et al. [15] found that 9/13 BCI users could separate left/right sources and that 7/9 performed better with binaural than with monaural stimulation. Each participant had their CI processor maps adjusted to equalize the loudness for the two ears. This study, and most others, also restricted head movements and this may have limited their performance, particularly in the monaural state. Beijen et al. [16] found that 5 BCI users localized more accurately than 5 UCI users. The initial phase (or turn) of participants’ head movements was used to characterize the response, as in other studies [14,17].

In this study we examined the use of two methods to compare the spatial hearing of children who were normally hearing (NH) with those who received UCI or BCI in early childhood. Because of the immaturity of binaural and spatial hearing in NH children [18,19], we evaluated children in two different age groups as well as a group of NH young adults. In two separate tasks, these children and adults were examined for lateral release (LR) and free-field sound localization acuity. LR is the improved recognition of a frontally presented target (signal) sound when a competing (masking) noise is moved from a front to a lateral position. We used the McCormick Toy Discrimination Test [20], a commercially available and widely used method with proven reliability [21] and familiar to the participants in our study. Single word noun stimuli were embedded in sentence form and both the intensity and spectral characteristics were roved to prevent discrimination using other than spatial cues. Like Beijen et al. [16] and Lovett et al. [14], we allowed free head movements and only tested CI users (in both groups) with processor maps to which they were fully accustomed.

We used similar tests to some of those used by Lovett et al. [14]. The children in that study were clinically and demographically heterogeneous and measures of LR were reported but not the speech-reception thresholds from which the measures of LR were computed. Nor were data for individual children reported. The present study recruited a more homogeneous group of UCI users and we report test results in more detail. However, the number of BCI using children was small and they formed a heterogeneous group. Their data should therefore be considered preliminary. The main aim was to compare the spatial hearing of NH with that of CI using children under ecologically valid conditions. We hypothesized that, firstly, NH listeners would perform more accurately on all tests than CI listeners and, secondly, UCI listeners would be unable to localize at better than chance levels.

## 2 Materials and methods

### 2.1 Listeners

Forty NH children were recruited through the ENT department at Queen's Medical Centre, Nottingham, were divided into two age groups (6–10 y.o., mean = 8.3 y.o., *n* = 26; 11–15 y.o., mean = 12.9 y.o., *n* = 14), to check for age-related changes in performance. Six NH adult listeners were recruited from research staff. All NH listeners were audiometrically normal (≤20 dBHL, 0.5–4 kHz inclusive, bilaterally [22]).

CI using children (bilateral, mean age = 8.8 y.o., *n* = 6; unilateral, mean age = 10.3 y.o., *n* = 12) were mostly prelingually deaf (Table 1) and were all fitted with Nucleus devices. UCI listeners (contralateral ears unaided and profoundly deafened) were more experienced device users (mean = 6.8 years; s.d. = 3.2) than BCI listeners (mean = 2.2 years; s.d. = 0.9). The BCI group contained two post-lingually deafened individuals who had a relatively short experience of deafness prior to implantation and who were also the oldest of the BCI sample tested. All CI users had stable electrode/pitch maps, at least 1 year's listening experience with their current configuration (UCI or BCI), full or near-full insertions, and no uncorrected visual impairment. They were contacted and recruited through the Nottingham and Birmingham Cochlear Implant Programmes. The processor and device(s) were confirmed to be functioning optimally immediately prior to testing. Of the BCI listeners, 3 had implants (simultaneously) inserted during a single surgical procedure and 3 had implants inserted during sequential procedures (Table 1). Experiments took place in a sound-attenuated and echo-damped chamber.

All listeners were native English speakers and participated in two experiments. Approval was received from the Nottingham Research Ethics Committee 1 and the Nottingham University Hospitals NHS Trust's Research and Development department.

### 2.2 Sound delivery

**Fig. 1:**
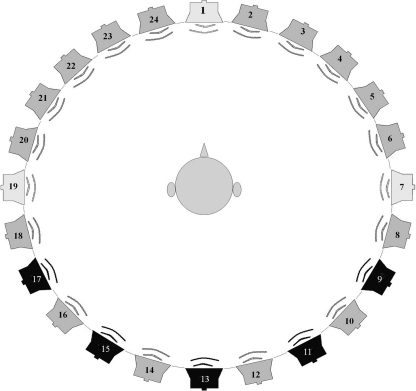
Loudspeaker ring. For Experiment 1, listeners faced Loudspeaker 1. Signals were delivered from Loudspeaker 1 (0°) and noise was delivered from Loudspeakers 1, 7 (+90°, right) and 19 (−90°, left). For Experiment 2, the listener faced Loudspeaker 13 and signals were delivered from Loudspeakers #9, #11, #13, #15 and #17. Video monitors were attached to the support poles of each of these loudspeakers.

Sounds were delivered by a loudspeaker ring (Fig. 1), developed at IHR, that had 24 individually calibrated (and #numbered), wide-range loudspeakers (Bose Acoustimass – cubes) mounted on aluminium poles. The poles were positioned around a dais, 3 m diameter, producing a 15° separation between the loudspeakers. Audio stimuli could be presented through an individual or any combination of loudspeakers, using digital to audio converters (Fostex VC-8) through a 24-channel interface (MOTU 2408).

## 3 Procedure

### 3.1 Experiment 1: lateral release (LR)

A chair was placed at the centre of the loudspeaker ring, 1.5 m from each loudspeaker. In front of the seated listener was a table displaying 14 Toy Test [23] toys. From Loudspeaker 1 (Fig. 1), a recording of a female talker said “Point to the …” followed by one of the toy names. Each toy had a matching toy sharing a similar vowel (e.g. ‘duck’ and ‘cup’) or diphthong. A correct identification was indicated by pointing or verbally identifying the target toy. In three noise conditions, pink (1/f) noise was played (60 dBA; measured at the child's head) from either the same loudspeaker as the target speech (Loudspeaker 1), or from Loudspeakers 7 (+90°) or 19 (−90°). Listeners performed each noise and toy name condition twice and the sequence of conditions was counterbalanced across listeners.

The target presentation level varied randomly between 49 and 57 dBA. Thresholds were determined using a two-phase adaptive staircase. In Phase 1, sound level decreased by 12 dB per step. The first incorrect response resulted in a reversal (the stimulus level increased) and the next correct response initiated Phase 2. In this ‘testing phase’, step sizes were reduced to 6 dB and a ‘2-down, 1-up’ adaptive rule [24] was used until six reversals occurred, the mean of which was threshold. LR was the mean threshold at 0° minus that at ±90°. Analysis used paired *t*-tests within groups. A one-way ANOVA between groups assessed differences in LR.

### 3.2 Experiment 2: localization acuity

Five loudspeaker poles (#9, #11, #13, #15, #17) were fitted with a 15″ colour flatscreen video monitor directly underneath the loudspeakers. The identical face of a talker was shown on each monitor. An inset picture (top left) showed a different Toy Test toy on each monitor. A synchronized audio stimulus, played from one, random loudspeaker, asked “Hello, what toy is this?” The visual stimulus was played, in four separate conditions, from 1–3 or all 5 video displays symmetrically arranged around Loudspeaker #13. The listener's task was to identify the active loudspeaker by naming the inset toy displayed on the coupled monitor. Each test condition had 30 trials. Condition 1S (1 loudspeaker, 1 monitor) was for familiarization. Conditions 2S (1 loudspeaker, 2 monitors, 120° separation), 3S (1 loudspeaker, 3 monitors, 60° separation), and 5S (1 loudspeaker, 5 monitors, 30° separation) tested sound localization accuracy through increasing levels of difficulty.

Listeners sat upright, fixating the monitor at Loudspeaker #13, but could move their head after the onset of the audio stimulus and were, in fact, observed to do so. Allowing head movements was part of the ‘ecological’ design of the experiment. The audio stimulus (6 s duration) intensity level was roved (59–67 dBA) to reduce use of level cues, and seven different spectral shaped variations of the talker's voice were also roved to reduce the use of monaural spectral cues. Feedback (either a verbal “well done” or “never mind, try again”) was given throughout the trials. Data were analysed using logistic regression. Differences between groups were tested using the likelihood ratio test statistic ‘lambda’ (Λ [25]), reported as −2 log (Λ) which, for small numbers of targets, is a more sensitive measure of localization than the traditional RMS error [26]. Data comparing localization decisions with chance performance, were further analysed using the *G*-test, a method for analyzing continguency tables based on a log-likelihood ratio and offering greater precision than the Pearson chi-square [27].

## 4 Results

### 4.1 Experiment 1: lateral release

Word discrimination thresholds in quiet (at 0°) showed no significant difference between the NH groups (mean thresholds 10.3–12.1 dBA; *F*(2,43) = 0.56, *p* = 0.58) or between the UCI and BCI groups (mean thresholds 35.6 and 33.0 dBA; *F*(1,16) = 0.49, *p* = 0.49). However, the NH groups performed better in quiet than the CI groups (*F*(2,61) = 135.91, *p* < 0.001).

**Fig. 2:**
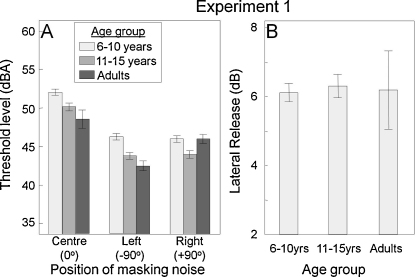
Normally hearing listeners. (A) Word discrimination thresholds in noise and (B) lateral release (LR). In Figs. 2–4, histogram bars are means and error bars are the standard error of the mean.

**Fig. 3:**
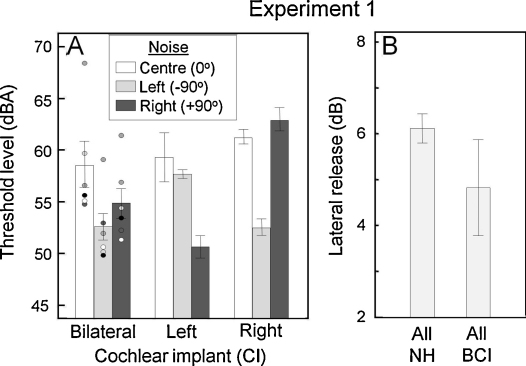
Listeners using cochlear implants (A) Word discrimination thresholds in noise for the bilateral (BCI). Data points show individual results, with different shading for each individual. For the unilateral listeners, ‘Left’ and ‘Right’ refer to the implanted ear. (B) LR for all normally hearing (NH) and BCI listeners.

Thresholds in noise are presented in Figs. 2 and 3. All listeners in both the NH (Fig. 2A) and CI (Fig. 3A) groups could perform the tasks. At 0° the NH groups had significantly lower thresholds than the CI groups (*F*(2,55) = 57.83, *p* < 0.001). Thresholds differed significantly (*F*(2,43) = 9.90, *p* < 0.001) between NH groups, with the adult group achieving the lowest mean threshold. However, there was no significant difference between the 6–10 y.o. and 11–15 y.o. children (*F*(1,38) = 2.96, *p* = 0.09). Among the CI listeners, there was no significant difference in thresholds between the UCI and BCI subgroups (*F*(1,16) = 1.63, *p* = 0.22).

When the noise masker was separated from the target and presented from ±90°, thresholds were generally (all NH and 5/6 BCI) reduced on both sides. For the NH group, no significant difference was found between sides (*t*(45) = 0.44, *p* = 0.67). However, in the BCI group, a small but significant threshold advantage for the left side was observed (*t*(5) = −2.53, *p* = 0.05). This was primarily attributable to one sequentially implanted listener who showed no LR when the noise was on the right side. A second sequentially implanted listener had elevated thresholds in all conditions. For UCI listeners, when the noise was presented from the same side as the CI, thresholds for most listeners were comparable to those seen with the noise at 0° and there was no LR. When the noise was presented from the side opposite the CI, thresholds were statistically comparable to those seen on the better (left) side of the BCI group and LR was comparable to that seen in the NH listener groups.

LR data for the NH and BCI groups are shown in Figs. 2B and 3B. As expected, LR was seen for all groups when the noise was spatially removed from the target (±90°). It was significant for the NH listeners (*t*(45) = −21.4, *p* < 0.001) and did not differ significantly between NH groups (*F*(2,43) = 0.34, *p* = 0.71) or between NH and BCI groups (*F*(1,50) = 2.18, *p* = 0.15; Fig. 3B). One sequentially implanted BCI listener lacked LR when the noise was on the right (second implanted) side, suggesting that target detection was being performed using the left ear only.

### 4.2 Experiment 2: localization

**Fig. 4:**
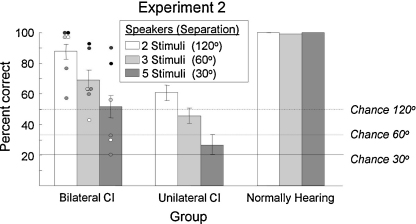
Localization acuity for the CI and NH groups. Chance levels indicate the likelihood of a randomly selected target being correct in each condition. Individual data points are shown for the Bilateral CI listeners.

The localization performance of all NH listeners was at or near ceiling on each of the three localization conditions (Fig. 4), demonstrating the simplicity of the task for this group. The accuracy of some BCI listeners across the three conditions was markedly poorer than the NH listeners, but was significantly (−2log(Λ) = 104.57, d.f. = 10, *p* < 0.001) better overall than that of UCI listeners. However, two post-lingually deafened, simultaneously implanted listeners scored highly (≥80%) for all conditions, including the most challenging one (5S). UCI listeners performed significantly above chance in all conditions (Condition 2S: *G* = 17.04, *p* < 0.001; 3S: *G* = 23.07, *p* < 0.001; 5S: *G* = 4.65, *p* = 0.031; all d.f. = 1) and one (of 3) post-lingually deafened UCI listener scored more highly than any of the other UCI listeners.

It may be noted that a purpose of separating the NH group by age was to search for developmental factors in the total age range of CI users that may have been confounded with their laterality and other properties of CI usage. As there were no developmental changes in the NH group for the main outcome measures: word discrimination, LR or localization scores (although the latter were at ceiling), it was safe to include the relatively wide age range of CI users into single groups.

## 5 Discussion

These results demonstrate that paediatric BCI listeners, unlike UCI listeners, can benefit when the noise occurs on either side of the head, thus improving their chances of detecting and using target speech in noisy environments. Although speech detection levels were elevated for BCI listeners, relative to NH listeners, their LR levels were comparable. BCI listeners also have significantly better localization acuity than UCI listeners, but do not perform as well as NH listeners. The best performing listeners for sound localization, among both the BCI and UCI groups, were deafened post-lingually.

Previous experiments have examined the same BCI listeners tested in ‘unilateral’ (i.e. one implant turned off) and ‘bilateral’ modes [28]. The testing of BCI listeners, acting as their own unilateral control, has the advantage of reducing variance, but introduces other interpretation difficulties. UCI listeners learn to use their implant for some months following implantation [29,30]. Assuming this also to be true of BCI listeners, the learning will have been to the cues received by both implants. If one is then temporarily disabled, the user may be at a disadvantage, in the unilateral mode, relative to an experienced UCI listener. In fact, we found here that UCI listeners performed above chance on the localization task. This may not be the case immediately after UCI switch-on, or immediately after unilateral switch-off in an experienced BCI listener. Testing an experienced UCI listener group in this study has allowed a direct, fair comparison between the spatial hearing of BCI and UCI listeners. It must be emphasised, however, that the BCI listeners tested here generally received their second (or simultaneous) implants at a later age than that at which the UCI listeners received their implants, and that the numbers in both groups of CI users were small while the heterogeneity of the BCI group, in particular, was large.

On the LR task, the UCI listeners improved when the noise was spatially separated from the target and directed towards the contralateral (non-implanted) ear. This indicates they were taking advantage of a relative decrease in the masking noise level on the side of the implant created by the acoustic ‘head shadow’ effect. BCI listeners were able to benefit from the same effect when the noise masker was directed toward either ear. But BCI listeners can also, in principle, benefit from binaural hearing, taking advantage of binaural unmasking and summation [31,32]. In this case, we may have expected enhanced LR for BCI listeners with the noise on either side, relative to that seen in the UCI listeners with the noise on the unimplanted side. However, in the BCI listeners tested here, no such enhancement was observed and performance was markedly inferior to NH listeners. There was thus no obvious benefit in the BCI listeners from central processing effects.

The performance of BCI listeners in the localization task confirmed [15,33,34] that acuity is better than UCI listening. NH listeners rely primarily on interaural time differences (ITDs) for localization in the horizontal plane [35]. However, the dominant cue for localization in BCI listeners appears to be interaural level differences (ILDs), with some CI listeners also being able to access envelope ITDs [36,37]. The main reason usually given for the relatively poorer localization of UCI listeners is that they are unable to use ILDs, and this conclusion is supported by findings that BCI listeners in unilateral mode perform at or near chance in localization tasks [34]. A previous study of sound localization [16] that examined both BCI and UCI child listeners found that UCI listeners could not localize sounds significantly above chance. Similar results have been found in adults [38,39], but Grantham et al. [30] found above chance UCI performance that was thought to be due to spectral information in the stimuli.

Listeners in this experiment were allowed to move their heads, in contrast to most other studies [10,15,30,31,33,34,38–43]. This was an attempt to simulate a more ecologically valid situation and thus provide information on how CI listeners perform in their daily environments. Our data show that experienced UCI listeners can perform at a level significantly above chance on this localization task. This level of performance was presumably facilitated by access to dynamic cues provided by head movements [44,45] and learning to use both stationary and dynamic intensity cues in the implanted ear.

As argued above, BCI listeners tested unilaterally, with little or no experience of dynamic cues, may also be disadvantaged relative to experienced UCI listeners. However, some of the experienced BCI listeners tested here localized at ceiling levels, and significantly better than the UCI listeners. While BCI listeners may have used distinctly binaural cues to achieve this level of localization, it is also possible that the second implant enabled them to scan their frontal field well on both sides of the midline using one implant, independently, on each side. Alternately, BCI listeners, like NH listeners, may use a combination of monaural and binaural cues in spatial hearing.

Although numbers were small, our data are consistent with the idea that the spatial hearing of simultaneously implanted BCI listeners is better than that of sequentially implanted BCI listeners. This difference may be due to the relative improvement in central processing obtained by simultaneous implant insertion. The BCI group included two simultaneously implanted listeners who were post-lingually deafened, had <6 months of deafness, then had >2years of BCI experience. This experience would have allowed learning-based plasticity of binaural unmasking [46], which may occur more slowly than other aspects of post-implantation learning [47]. The small sample of BCI listeners, and its heterogeneity, suggests caution in the interpretation of inter-implant delay from this study alone, but other recent literature has demonstrated that shorter (<2years) inter-implant delays are associated with better outcomes [48,49].

One challenge of testing children is creating an engaging task. This LR experiment used the Toy Test [50], also used for assessing spatial hearing in CI listeners by Lovett et al. [14]. The Toy Test has several advantages in clinical practice: it can be used in children as young as two, it has test–retest reliability [21], it has been extensively used as a pediatric audiological test, it can be easily tailored to the vocabulary of the individual child, and it may be used, as here, in the free-field. Within the local cochlear implant program the Toy Test is used regularly to assess auditory thresholds in quiet, so the CI listeners were familiar with the methodology, reducing the need for repeated testing. The LR in the NH groups demonstrated in this experiment, using the Toy Test, was similar in magnitude to reported data (e.g. [14,51]).

The spatial hearing of profoundly hearing impaired listeners using BCI is better than that of users of a single CI, but remains markedly poorer than that of NH listeners. In future experiments we will examine whether training CI listeners can narrow this performance gap.

## Figures and Tables

**Table 1:** Demographics of cochlear implant using children. Onset age is listed as Congen(ital) or in months (m). For bilateral implantation, Age is stated for the first/second implant, and surgery was either sequential or simultaneous. Linguistic development at first implantation is assessed as either pre- or post-lingual.

A						
Bilateral ID	Age (yrs)	Onset	Age @ CI (m)	Surgery	Aetiology	Pre/Post Lingual
101	6	Congen	20/54	Seq	Unknown	Pre
112	6	Congen	41	Sim	Unknown	Pre
116	11	Congen	42/130	Seq	Connexin 26	Pre
117	8	Congen	36/63	Seq	Waadenburg	Pre
122	11	108 m	111	Sim	Meningitis	Post
123	11	84 m	90	Sim	Meningitis	Post

B						
Unilateral ID	Age (yrs)	Onset	Age @ CI (m)	CI Side	Aetiology	Pre/Post Lingual
102	10	Congen	23	Left	Unknown	Pre
103	12	Congen	36	Right	Unknown	Pre
104	12	Congen	43	Right	Unknown	Pre
105	16	Congen	60	Right	Unknown	Pre
107	8	11 m	26	Right	Meningitis	Post
109	7	?	62	Right	Unknown	Post
110	12	Congen	53	Right	Unknown	Pre
111	11	Congen	59	Left	Unknown	Post
113	10	Congen	27	Right	Connexin 26	Pre
114	12	Congen	38	Right	Connexin 26	Pre
115	8	Congen	30	Left	Unknown	Pre
118	8	Congen	22	Right	Unknown	Pre
